# Editorial: Epigenetic Regulation in Cardiovascular Diseases

**DOI:** 10.3389/fcvm.2021.831851

**Published:** 2022-01-11

**Authors:** Indulekha C. L. Pillai, Suowen Xu, Christoph D. Rau, Zhihua Wang

**Affiliations:** ^1^Stem Cells and Regenerative Biology Laboratory, School of Biotechnology, Amrita Vishwa Vidyapeetham, Kollam, India; ^2^Institute of Endocrine and Metabolic Diseases, The First Affiliated Hospital of USTC, University of Science and Technology of China, Hefei, China; ^3^Computational Medicine Program and Department of Genetics, The University of North Carolina at Chapel Hill, Chapel Hill, NC, United States; ^4^Shenzhen Key Laboratory of Cardiovascular Disease, Fuwai Hospital Chinese Academy of Medical Sciences, Shenzhen, China; ^5^State Key Laboratory of Cardiovascular Disease, National Center for Cardiovascular Disease, Fuwai Hospital, Chinese Academy of Medical Sciences and Peking Union Medical College, Beijing, China

**Keywords:** epigenetics, cardiovascular disease, DNA methylation, histone modification, non-coding RNA

Cardiovascular disease (CVD) is the leading cause of death globally. Progress in the diagnosis, prevention, and treatment of CVD is contingent on the advancement of our knowledge to explain the complex pathophysiology underlying CVD in which gene expression re-programming plays a fundamental role. Emerging evidence highlights the impact of epigenetic regulation on the transition of gene expression patterns from physiological to pathological states. Epigenetics, originally defined as stably heritable phenotypes resulting from changes in a chromosome without alterations in the DNA sequence, is now more broadly understood to encompass any modification to DNA structure or function that influences phenotypes related to development or disease other than an actual change to the sequence. The epigenetic environment of a gene is mostly determined by DNA methylation, histone modifications, and chromatin remodeling. Various writers, readers, and erasers for different epigenetic marks have been discovered, and their dysfunction tightly correlates with the development of CVD. Research elucidating epigenetic regulations in this field have, in turn, promoted novel drug discoveries to treat CVD. The identification of novel epigenetic players in CVD and how they act to fine-tune molecular processes would help expand our understanding of the complexity of cardiovascular pathophysiology. In the current Research Topic, we have collected 16 high-quality studies that cover promising, recent, and novel research trends in the epigenetic regulation of CVD.

Using an integrated approach with gene expression and DNA methylation profiles, Zhang et al. identify novel biomarkers, including FN1, PTEN, and POLR3A, for coronary artery disease. Li et al. find that the co-occurrence of chronic hepatitis B and fibrosis is associated with a decrease in hepatic global DNA methylation levels in patients with non-alcoholic fatty liver disease. A review by Xu et al. summarize the roles and mechanisms of DNA methylation in vascular aging and related diseases. Furthermore, Ju et al. find that diabetic cardiomyopathy is accompanied by global changes in m^6^A RNA modification, possibly due to the alteration of FTO expression.

By combining RNA-seq with ChIP-seq, Wang et al. systematically investigated the genome-wide profiles of five histone marks (H3K27ac, H3K9ac, H3K4me3, H3K9me3, and H3K27me3) in the early stage of myocardial infarction, and clarify a H3K27ac-related gene expression pattern associated with angiogenesis. Greenway et al. profile histone modification patterns in aortic tissues during abdominal aortic aneurysms formation in two distinct mouse models, angiotensin II infusion and calcium chloride overload. A review by Li et al. links epigenetic mechanisms with oxidative stress-induced ferroptosis in CVD.

By screening patients with atherosclerosis, Gao et al. identify miR-135a-5p as a protective factor against atherosclerosis development by directly targeting JAK2. Wang et al. identify novel non-coding RNAs, especially a class of snoRNAs, being dynamically associated with a H3K27 methyltransferase EZH2 at the early phase of cardiac hypertrophy. A review by Saadat et al. summarizes emerging evidence that non-coding RNAs, including miRNAs, circRNAs and lncRNAs, modify the TGF-β/Smad Signaling during the progression of cardiac fibrosis.

Wei et al. sequenced individuals with or without hypertension from an ethnic minority of China, and finds that haplotypes of CYP17A1 and ATP2B1 are correlated with hypertension risks. Yang et al. find that GRB10 rs1800504 polymorphism is associated with the risk of coronary heart disease in patients with type 2 diabetes mellitus. Liu et al. unveil a causal effect of statin use on type 2 diabetes and related traits through epigenetic mechanisms, specifically, DNA methylation at cg06500161 of ABCG1. Kashyap et al. find that antiretroviral drug treatment significantly reduces acetylation at H3K9 and H3K27 and promotes methylation at H3K9 and H3K27 possibly through regulating the expression of SIRT1, SUV39H1, and EZH2. A review by Chen et al. presents an updated summary on the roles of HDACs and HDAC inhibitors in vascular dysfunction with an emphasis on therapeutic targets and agents in atherosclerotic cardiovascular diseases. Finally, Ahmed et al. develops a network-based approach using a novel algorithm, IVI (Integrated Value of Influence), to dissect genes with important roles in cardiovascular disease and chronic kidney disease.

Taken together, the present Research Topic represents an important source of up-to-date information, covering most aspects of epigenetics in the CVD field with a broad view from basic research to clinical trials and from fundamental mechanisms to precision medicine. More comprehensive knowledge based on these discoveries may bring about new diagnostic and therapeutic approaches.

## Author Contributions

All authors listed have made an equal, substantial, direct, and intellectual contribution to the work and approved it for publication.

## Conflict of Interest

The authors declare that the research was conducted in the absence of any commercial or financial relationships that could be construed as a potential conflict of interest.

## Publisher's Note

All claims expressed in this article are solely those of the authors and do not necessarily represent those of their affiliated organizations, or those of the publisher, the editors and the reviewers. Any product that may be evaluated in this article, or claim that may be made by its manufacturer, is not guaranteed or endorsed by the publisher.

